# Utilisation of cancer screening services by disabled women in Chile

**DOI:** 10.1371/journal.pone.0176270

**Published:** 2017-05-01

**Authors:** Dikaios Sakellariou, Elena S. Rotarou

**Affiliations:** 1 School of Healthcare Sciences, Cardiff University, Cardiff, United Kingdom; 2 Department of Economics, University of Chile, Santiago, Chile; Rudjer Boskovic Institute, CROATIA

## Abstract

**Background:**

Research has shown that women with disabilities face additional challenges in accessing and using healthcare services compared to non-disabled women. However, relatively little is known about the utilisation of cancer screening services for women with disabilities. This study addresses this gap by examining the utilisation of the Papanicolaou test and mammography for disabled women in Chile.

**Methods:**

We used cross-sectional data, taken from a 2015 nationally-representative survey. Initially, we employed logistic regressions to test for differences in utilisation rates for the Papanicolaou test (66,281 observations) and the mammogram (35,294 observations) between disabled and non-disabled women. Next, logistic regressions were used to investigate the demographic, socioeconomic, and health-related factors affecting utilisation rates for cancer screening services for disabled women (sample sizes: 5,823 observations for the Papanicolaou test and 5,731 observations for the mammogram).

**Results:**

Disabled women were less likely to undergo screening tests than non-disabled women. For the Papanicolaou test and mammography, the multivariable regression models showed that living in rural areas, having higher education, being affiliated with a private health insurance company, giving a good health self-assessment score, and being under medical treatment for other illnesses were associated with higher utilisation rates. On the other hand, being single, inactive with regard to employment, and having a better income were linked with lower utilisation. While utilisation rates for both disabled and non-disabled women have increased since 2006, the utilisation disparity has slightly increased.

**Conclusions:**

This study shows the influence of various factors in the utilisation rates of preventive cancer screening services for disabled women. To develop effective initiatives targeting inequalities in the utilisation of cancer screening tests, it is important to move beyond an exclusively single-disease approach and acknowledge the complexity of the patient population.

## Introduction

Access to and effective usage of healthcare services are basic human rights. Often, however, these rights are not distributed equitably: research indicates that disabled people can face several challenges in their efforts to use healthcare services.[1] In this article, we examine utilisation rates of the Papanicolaou test (Pap test) and mammography for women with and without disabilities in Chile.

Most of the existing evidence suggests that women with disabilities have lower utilisation rates and worse access to preventive health services compared to women without disabilities.[2–5] The majority of these studies are small-scale studies, which although they give important insights into the experiences of women as they navigate the healthcare system, they do not allow any conclusions regarding utilisation of preventive services at a population level. There is a notable lack of population-based data, on how gender, age, and socioeconomic variables jointly intersect to affect the utilisation of healthcare services for women with disabilities, especially in South America. It is important to know which are the factors that affect the utilisation of preventive services, so that policies and targeted interventions can be implemented to address any inequalities.

Our aim in this article is to examine the utilisation of preventive cancer screening services for women with and without disability in Chile, and explore the factors influencing utilisation of such services by disabled women. Chile has some of the highest cancer screening levels in South America [6, 7] but also a highly unequal healthcare system, in terms of its access.[8, 9] Based on cross-sectional data from the National Socioeconomic Characterisation Survey, we looked at the intersections between disability and utilisation of two preventive services for women: the Pap test, which screens for cervical cancer, and the mammogram, which screens for breast cancer.

While Chile’s health indicators are among the best in South America and are similar to those of highly-industrialised countries, the country suffers from inequalities in access to healthcare, and insufficient protection from health risks (this is mostly the case for people who are affiliated with the public healthcare provider, who often face difficulties both regarding financial protection and access to timely attention).[10] Healthcare services are provided mainly through the public health provider (FONASA) and the thirteen available private insurance companies (ISAPREs). FONASA covers more than two thirds of the population, while about 18% of Chileans are covered by ISAPREs. The contract premium for ISAPREs is determined by sex, age, and risk, a fact that often excludes women of reproductive age, the young, and the elderly. This has led to the stratification of access to healthcare, so that people with lower financial resources access the underfunded and overburdened public health system.[9]

As part of the National Commission for Cancer, the National Programme for Cervical Cancer and the National Programme for Breast Cancer were established in 1987 and 1995 respectively, aiming to coordinate diagnostic, treatment, evaluation, and monitoring activities.[11] The Pap test and the mammogram have been the pillars of the screening programmes for these two types of cancers. Both cervical and breast cancer are now covered under the AUGE-GES plan (explicit guarantees for people in a group of eighty prioritised pathologies, independent of their ability to pay for health services), which was a significant part of the 2000 health reform in Chile, aimed at increasing equity in healthcare. These initiatives have enabled women to access diagnostic and treatment services, contributing to further increased national screening coverage.[6, 12, 13]

Currently, incidence of breast cancer in Chile is 23.4/100,000 women, making it the first most common cancer for women; incidence of cervical cancer is 8.3/100,000 women, making it the fourth most common cancer.[14] The latest available data from 2013 show that screening coverage for cervical cancer reached 57.9% of women aged 20–69 years; the screening coverage for breast cancer reached 32.8% of women aged between 50 and 69 years.[15] While these data place Chile as the country with the highest percentage of cervical cancer screening in Latin America,[6] and on par with the median utilisation rate of countries belonging to the Organisation for Economic Cooperation and Development (OECD), [15] breast cancer screening is still low; the median utilisation rate for a mammography for OECD countries in 2013 was 58%, much higher than the reported 32.8% in Chile.[15]

## Methods

### Study aim and methods

The aim of the study was to examine the utilisation rates of the Pap test and the mammogram for women with and without disability in Chile, and the factors influencing utilisation for disabled women. We also looked at utilisation rates between disabled and non-disabled women during the 2006–2015 period, in order to examine whether utilisation changed during that time. Since the national guidelines recommend a Pap test every three years from ages 25 to 64 and one mammogram every two years between the ages of 50 and 74,[16, 17, 18] this study includes women between 25 and 65 years of age for the investigation of utilisation rates of the Pap test, and women between the ages of 50 and 75 for the mammogram.

Our study was based on data available from the 2015 National Socioeconomic Characterisation Survey (Encuesta Nacional de Caracterización Socioeconómica–CASEN), conducted by the Ministry of Social Development of the Government of Chile. The results of the CASEN survey are anonymised and are freely available, as is the methodology, from the website of the Ministry of Social Development of the Chilean government.[19] The 2015 CASEN survey covered 83,887 households– 266,968 people–across the fifteen regions and 324 counties of Chile. The survey was performed as a personal interview–lasting, on average, 47 minutes for a household of four people–from November 2^nd^ 2015 until January 31^st^ 2016. It included seven modules: registry of residents, education, employment, income, health, residents, and housing.

The main analysis involved a cross-sectional comparison of disabled and non-disabled women regarding their utilisation rates of the Pap test and mammogram. Additionally, a supplemental longitudinal analysis was performed to examine differences between these two groups since 2006 (for the Pap test) and since 2011 (for the mammogram). All analyses were performed using STATA Version SE 11.2. Firstly, logistic regressions were used to investigate any possible difference in the utilisation rates of the Pap test and mammogram between disabled and non-disabled women. Secondly, logistic regressions were performed, after controlling for demographic, socioeconomic, and health-related variables, in order to investigate the impact of these factors on the utilisation rates of screening services for disabled women in Chile.

### Data and variables

The sample sizes of our study included 66,281 women (8.7% of whom were disabled), aged 25–65, for the analysis of utilisation rates for the Pap test, and 35,294 women (16.2% of whom were disabled), aged 50–75, for the mammogram. Women were asked whether they have done the Pap test or a mammogram in the last three years; the answers were ‘no’ (this answer was left as ‘no’), ‘yes, during the last year’, ‘yes, more than a year ago and less than two’, and ‘yes, more than two years ago and less than three’ (the last three answers were recoded under the category ‘yes’).

The demographic, socioeconomic, and health-related variables that were used as controls in our study included the following: a) *geographical location*: urban / rural; b) *age groups*: 25–34 / 35–49 / 50–65 (for the Pap test) and 50–64 / 65–75 (for the mammogram); c) *civil status*: married / living with or in a relationship / separated, divorced or annulled / widowed / single; d) *indigeneity*: not indigenous / indigenous (includes people from nine state-recognised indigenous groups); e) *equalised income* (log): household income divided by square root of household size (square root equivalence scale); f) *education*: years of schooling; g) *employment*: employed / unemployed / inactive; h) *housing*: acceptable / substandard / unacceptable (classification indicated housing quality: good building materials and sanitation were characteristics of an acceptable type of housing, deficient sanitation or substandard but repairable building materials were features of a substandard type of housing, while all dwellings with irreparable building materials–independent of sanitation–fell under the category of unacceptable type of housing); i) *health self-assessment*: scores 1–2 = ‘bad’ / scores 3–5 = ‘neither good nor bad’ / scores 6–7 = ‘good’; j) *health provider*: FONASA (public) / Armed forces / ISAPRE (private) / out-of-pocket; and k) *medical treatment*: no / yes (included treatment for 22 illnesses, such as hypertension, diabetes, depression, and various types of cancer).

In the CASEN survey, disability was self-reported and defined on the basis of Law No. 20.422 of 2010, which defines a disabled person as one that has one or more physical, mental (either due to a psychological or intellectual cause) or sensory impairment, and who in an effort to interact with his or her environment, finds that his or her full and effective participation in society on an equal basis with others is impeded or restricted. [19]

## Results

**Fig 1:**
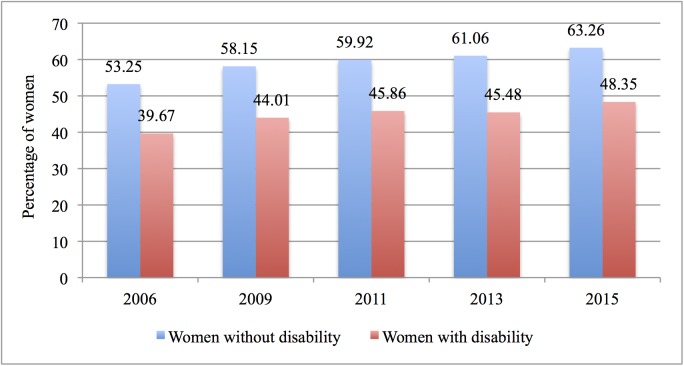
Utilisation of Pap test by women with and without disability (2006–2015).

**Fig 2:**
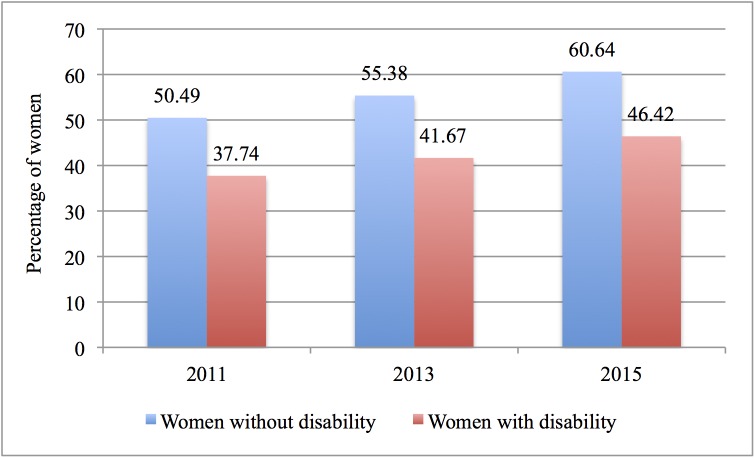
Utilisation of mammogram by women with and without disability (2011–2015). Note to Fig 2: The question on mammogram was included in the CASEN survey in 2011.

Figs 1 and 2 show that in Chile fewer disabled women undergo both tests, compared to women without a disability. The differences in percentages are statistically significant, with the exception of the period between 2011 and 2013 for the Pap test for disabled women.

As it can be seen from the above figures, the utilisation rates of preventive services for both groups have been increasing.

Table 1 shows the reasons disabled and non-disabled women give about not undergoing the Pap test or mammography, according to the 2015 CASEN survey.

**Table 1:** Reasons for not undergoing Pap test and mammography for women with and without disability in Chile.

Reasons	Papanicolaou test Women’s age: 25–65				Mammography Women’s age: 50–75			
	Women without disability		Women with disability		Women without disability		Women with disability	
	n	%	n	%	n	%	n	%
Do not know where to do it	188	0.58	34	0.57	200	0.92	48	0.88
Test scares them or disgusts them	2,543	7.85	423	7.13	1,931	8.84	402	7.38
Forget to do it	3,478	10.73	418	7.04	2,759	12.63	419	7.70
Do not believe they need it	8,024	24.76	1,599	26.94	4,516	20.67	1,422	26.12
Do not know this test	313	0.97	53	0.89	110	0.50	53	0.97
Did not know that they have to do it	1,081	3.34	160	2.70	883	4.04	204	3.75
Hours at the clinic do not suit them	233	0.72	23	0.39	224	1.03	29	0.53
Do not have time	3,260	10.06	266	4.48	2,617	11.98	262	4.81
Unable to schedule an appointment	919	2.84	160	2.70	1,642	7.51	343	6.30
Do not have money	260	0.80	49	0.83	601	2.75	104	1.91
Test does not apply to them	8,802	27.16	2,025	34.12	3,666	16.78	1,390	25.53
Other reason	3,306	10.20	725	12.22	2,703	12.37	769	14.12
Observations	32,407		5,935		21,852		5,445	

Note: Differences for both Pap test and mammography are statistically significant with *p*<0.000.

A high proportion of disabled and non-disabled women answered that the Pap test and the mammogram did not apply to them; taking into account that these women were within the age range recommended for taking these tests, it is not quite clear why women gave this answer. It is, therefore, important to address possible issues of misinformation, such as the age that women need to do these screening tests or risk factors for developing breast or cervical cancer. A high percentage of women also answered that they did not undergo the tests because they did not believe they needed it, did not have time, or because they forgot to do it.

Table 2 presents a comparison of demographic, socioeconomic, and health-related characteristics of Chilean disabled women with regards to undertaking the Pap test and mammography.

**Table 2:** Characteristics of disabled women and their undertaking of Pap test and mammography.

Parameter	Pap test (N = 5,823)			Mammography (N = 5,731)		
	No	Yes	*p* value	No	Yes	*p* value
**Geographical location**						
Urban	1,345 (79.02%)	3,162 (76.73%)	*p* = 0.057	1,795 (77.07%)	2,623 (77.10%)	*p* = 0.979
Rural	357 (20.98%)	959 (23.27%)		534 (22.93%)	779 (22.90%)	
**Age groups**						
25–34	256 (15.04%)	432 (10.48%)	*p*<0.000	-	-	*p*<0.000
35–49	441 (25.91%)	1,180 (28.63%)		-	-	
50–64	1,005 (59.05%)	2,509 (60.88%)		1,008 (43.28%)	2,246 (66.02%)	
65–75	-	-		1,321 (56.72%)	1,156 (33.98%)	
**Civil status**						
Married	483 (28.38%)	1,727 (41.91%)	*p*<0.000	885 (38.00%)	1,599 (47.00%)	*p*<0.000
Living with or in a relationship	195 (11.46%)	636 (15.43%)		172 (7.39%)	311 (9.14%)	
Separated, divorced or annulled	211 (12.40%)	633 (15.36%)		283 (12.15%)	483 (14.20%)	
Widowed	95 (5.58%)	286 (6.94%)		482 (20.70%)	512 (15.05%)	
Single	718 (42.19%)	839 (20.36%)		507 (21.77%)	497 (14.61%)	
**Indigeneity**						
Not indigenous	1,498 (88.01%)	3,663 (88.89%)	*p* = 0.340	2,072 (88.97%)	3,053 (89.74%)	*p* = 0.348
Indigenous	204 (11.99%)	458 (11.11%)		257 (11.03%)	349 (10.26%)	
Equalised income (mean, sd)*						
	247,156 (280,907)	245,872 (263,876)	*p*<0.000	252,236 (217,024)	277,016 (291,987)	*p*<0.000
**Education (mean, sd)**						
	7.56 (5.12)	8.91 (4.39)	*p*<0.000	6.51 (4.34)	7.77 (4.49)	*p*<0.000
**Employment**						
Employed	424 (24.91%)	1,454 (35.28%)	*p*<0.000	351 (15.07%)	816 (23.99%)	*p*<0.000
Unemployed	48 (2.82%)	124 (3.01%)		23 (0.99%)	51 (1.50%)	
Inactive	1,230 (72.27%)	2,543 (61.71%)		1,955 (83.94%)	2,535 (74.51%)	
**Housing**						
Acceptable	1,387 (81.49%)	3,378 (81.97%)	*p* = 0.911	1,910 (82.01%)	2,885 (84.80%)	*p* = 0.016
Substandard	297 (17.45%)	701 (17.01%)		395 (16.96%)	492 (14.46%)	
Unacceptable	18 (1.06%)	42 (1.02%)		24 (1.03%)	25 (0.73%)	
**Health self-assessment**						
Bad	479 (28.14%)	970 (23.54%)	*p*<0.000	717 (30.79%)	958 (28.16%)	*p* = 0.085
Neither good nor bad	813 (47.77%)	2,186 (53.05%)		1,226 (52.64%)	1,878 (55.20%)	
Good	410 (24.09%)	965 (23.42%)		386 (16.57%)	566 (16.64%)	
**Health provider**						
FONASA	1,564 (92.16%)	3,750 (91.26%)	*p* = 0.057	2,212 (95.22%)	3,114 (91.70%)	*p*<0.000
Armed forces	16 (0.94%)	47 (1.14%)		26 (1.12%)	48 (1.41%)	
ISAPREs	82 (4.83%)	254 (6.18%)		52 (2.24%)	197 (5.80%)	
Out-of-pocket	35 (2.06%)	58 (1.41%)		33 (1.42%)	37 (1.09%)	
**Medical treatment**						
No	678 (39.84%)	1,318 (31.98%)	*p*<0.000	555 (23.83%)	681 (20.02%)	*p* = 0.001
Yes	1,024 (60.16%)	2,803 (68.02%)		1,774 (76.17%)	2,721 (79.98%)	

* Income is presented in Chilean pesos (1USD = 661 Chilean pesos, March 2017).

Initially, logistic regressions were performed to examine whether disabled women were more or less likely to undergo any of these tests compared to non-disabled women. There were 66,281 observations (out of which, 5,793 involved disabled women) for the Pap test, and 35,294 observations (out of which, 5,707 involved disabled women) for the mammogram. The results showed that disabled women were 1.4 times less likely than non-disabled women to do a Pap test (adjusted odds ratios: OR = .698, with a 95% confidence interval: CI = .654-.746) and 1.3 times less likely to do a mammogram (adjusted odds ratios: OR = .771, with a 95% confidence interval: CI = .723-.823). The results were statistically significant with *p* < 0.000.

Table 3 presents the results of logistic regressions for disabled women regarding factors influencing their undergoing or not a Pap test. Model (1) includes various demographic variables, Model (2) adds a range of socioeconomic characteristics, while Model (3) includes all the previous variables plus health-related variables.

**Table 3:** Results of logistic regression using *Papanicolaou test* as dependent variable (adjusted odds ratios).

Variables	Model (1)		Model (2)		Model (3)	
	OR	95% CI	OR	95% CI	OR	95% CI
**Geographical location (urban as reference)**						
Rural	1.081	.937–1.248	1.228**	1.053–1.433	1.215*	1.040–1.419
**Age groups (25–34 as reference)**						
35–49	1.223*	1.003–1.491	1.277*	1.041–1.566	1.241*	1.010–1.526
50–65	.999	.830–1.204	1.152	.948–1.399	1.076	.880–1.315
**Civil status (married as reference)**						
Living with or in a relationship	.896	.738–1.087	.851	.699–1.035	.863	.708–1.052
Separated, divorced, annulled	.844	.700–1.017	.665***	.547-.809	.658***	.540-.801
Widowed	.872	.676–1.125	.840	.648–1.089	.838	.645–1.088
Single	.325***	.280-.376	.323***	.277-.376	.326***	.279-.380
**Indigeneity (not indigenous as reference)**						
Indigenous	.920	.766–1.104	.930	.772–1.119	.932	.773–1.123
**Equalised income (log)**			.802***	.733-.877	.807***	.735-.887
**Education (years)**			1.055***	1.041–1.070	1.054***	1.038–1.069
**Employment (employed as reference)**						
Unemployed			.701	.488–1.008	.711	.493–1.024
Inactive			.608***	.527-.701	.603***	.522-.697
**Housing (acceptable as reference)**						
Substandard			.975	.828–1.148	.983	.834–1.158
Unacceptable			1.077	.602–1.926	1.144	.638–2.050
**Health self-assessment (“bad” as reference)**						
Neither good nor bad					1.384***	1.198–1.598
Good					1.389***	1.161–1.662
**Health provider (FONASA as reference)**						
Armed forces					1.155	.633–2.107
ISAPRE					1.025	.768–1.368
Out-of-pocket					.796	.508–1.247
**Medical treatment (no as reference)**						
Yes					1.414***	1.236–1.618
**Observations**	5823		5810		5793	
**Pseudo R^2**	0.0419		0.0624		0.0684	
**Chi^2**	294.58		438.15		478.78	
**Prob>Chi^2**	0.0000		0.0000		0.0000	
**McFadden R2**	0.039		0.058		0.062	
**Deviance**	6741.799		6582.173		6520.941	
**AIC**	6759.799		6612.173		6562.941	
**BIC**	6819.826		6712.183		6702.893	

* *p* < 0.05.

** *p* < 0.01.

*** *p* < 0.001.

The likelihood ratio chi-squares with a Prob>chi2 = .000 of all the models indicate that they are statistically significant, as compared to the null model with no predictors.

Due to a higher Mac Fadden *R*^2^, and lower deviance, and AIC and BIC values, Model (3) provides a better fit than the previous two models. There was no collinearity affecting the results, with mean variance inflation factor (VIF) of 1.39.

Regarding Model (3), disabled women in the 35–49 age group were 1.2 times more likely to do the test than women in the 25–34 age group. Single disabled women were more than three times less likely to do the test, compared to married women. Higher income decreased the probability of disabled women undergoing the test, while more years of education and living in rural areas increased such probability. Regarding employment status, inactive disabled women were 1.7 times less likely to do the test. Also, disabled women that gave a ‘neither good nor bad’ or a ‘good’ score to their health were 1.4 times more likely to do the test, compared to women who characterised their health as ‘bad’. Finally, disabled women who were under some kind of medical treatment were 1.4 times more likely to undergo a Pap test.

Table 4 presents the results of logistic regressions for disabled women regarding factors influencing their undergoing or not a mammography.

**Table 4:** Results of logistic regression using *mammography* as dependent variable (adjusted odds ratios).

Variables	Model (1)		Model (2)		Model (3)	
	OR	95% CI	OR	95% CI	OR	95% CI
**Geographical location (urban as reference)**						
Rural	.958	.839–1.094	1.133	.982–1.306	1.120	.970–1.293
**Age groups (50–64 as reference)**						
65–75	.384***	.343-.431	.426***	.379-.480	.411***	.364-.463
**Civil status (married as reference)**						
Living with or in a relationship	.873	.708–1.077	.880	.712–1.088	.906	.732–1.121
Separated, divorced, annulled	.829*	.697-.987	.755**	.629-.905	.744**	.619-.893
Widowed	.741***	.634-.865	.763**	.651-.894	.758**	.646-.889
Single	.487***	.417-.568	.484***	.414-.566	.495***	.423-.580
**Indigeneity (not indigenous as reference)**						
Indigenous	.899	.752–1.074	.950	.793–1.138	.964	.803–1.157
**Equalised income (log)**			.962	.881–1.050	.939	.857–1.028
**Education (years)**			1.045***	1.031–1.060	1.039***	1.024–1.054
**Employment (employed as reference)**						
Unemployed			.849	.504–1.430	.805	.476–1.360
Inactive			.740***	.636-.861	.719***	.620-.838
**Housing (acceptable as reference)**						
Substandard			.812*	.693-.951	.818*	.697-.959
Unacceptable			.751	.419–1.346	.783	.436–1.406
**Health self-assessment (“bad” as reference)**						
Neither good nor bad					1.146*	1.009–1.301
Good					1.145	.962–1.363
**Health provider (FONASA as reference)**						
Armed forces					1.238	.747–2.050
ISAPRE					1.952***	1.395–2.730
Out-of-pocket					.711	.435–1.162
**Medical treatment (no as reference)**						
Yes					1.470***	1.279–1.690
**Observations**	5731		5719		5707	
**Pseudo R^2**	0.0492		0.0594		0.0662	
**Chi^2**	381.15		458.72		510.48	
**Prob>Chi^2**	0.0000		0.0000		0.0000	
**McFadden R2**	0.047		0.056		0.061	
**Deviance**	7361.614		7267.739		7198.916	
**AIC**	7377.614		7295.739		7238.916	
**BIC**	7430.843		7388.861		7371.905	

* *p* < 0.05.

** *p* < 0.01.

*** *p* < 0.001.

All models are statistically significant (Prob>chi2 = .000). A higher Mac Fadden *R*^2^, and lower deviance, and AIC and BIC values, show that Model (3) provides a better fit than Models (1) and (2). There were no collinearity issues: the mean VIF was 1.17.

Disabled women in the 65–75 age range were 2.4 times more likely to do a mammogram, compared to women in the 50–64 age group. Disabled women in most civil statuses other than ‘married’ were less likely to do a mammogram, with single disabled women being twice less likely to do so. Disabled women with more years of education were more likely to undertake the mammogram, while inactive women and women living in substandard type of housing were less likely. Moreover, disabled women who reported their health as being ‘neither good nor bad’ were more likely to do the test than women who assessed their health as ‘bad’. Disabled women affiliated with a private health insurance company (ISAPRE) were twice more likely to do the test, compared to disabled women affiliated with FONASA. Finally, disabled women that underwent some kind of medical treatment were 1.5 times more likely to do a mammogram.

## Discussion

The aim of this study was to examine the utilisation of preventive cancer screening services for women with and without disability in Chile, and the factors influencing utilisation of such services by disabled women. Our results showed that disabled women in Chile were less likely than non-disabled women to undergo preventive screening tests. The study found that disabled women faced a combination of factors (demographic, socioeconomic, and health-related) that interacted with each other and affected the utilisation of services.

Disabled women with private health insurance (ISAPRE) were more likely to undergo a mammogram than women with public health insurance (FONASA); this result agrees with previous research showing that the type of insurance is predictive of healthcare access and likelihood of receipt of mammograms.[20, 21] On the other hand, while financial limitations are often reported as one of the main reasons why disabled women do not access preventative services,[22] our study shows that this is not necessarily the case; a very small percentage of disabled women in our study mentioned financial reasons for not doing the Pap test (0.8%) or the mammogram (1.9%). The reason behind this might be the introduction of these cancer screening procedures into the AUGE-GES system that have made the Pap test and mammography more accessible financially.

Furthermore, disabled women who were already on medical treatment for other illnesses were also more likely to undergo both preventive health tests, an indication of how people in general may become more health-conscious after or during a certain illness. Research has shown how chronic diseases are significant predictors for participation in preventive health check-ups.[23] Finally, this study also showed that single disabled women were three times less likely to undergo a Pap test, and twice less likely to undergo a mammogram, compared to married women. Various studies have explored the issue of single older women with disability being at risk due to lower rates of cancer screening.[24] The underuse of cancer screening services in this group indicates the need to identify specific barriers to low utilisation.

Our findings also show that since 2006 the utilisation disparity between disabled and non-disabled women has slightly increased; in 2015, it reached almost 15 percentage points for the Pap test, and more than 14 percentage points for the mammogram, despite the establishment of the AUGE-GES explicit guarantees system. While utilisation rates have actually increased for both groups, the slight increase in the utilisation gap is worrying. It is important to address this issue since disabled women are at a higher risk of late-stage cancer than women without disabilities, due to their lower utilisation rates of preventive services.[25]

Research has evidenced how access to cancer screening services can be compromised due to the presence of pre-existing disability.[2, 26, 27] This is due to a combination of personal, interpersonal, and structural factors, which can include understanding of the importance of screening, having a supporting social network, availability of accessible health facilities, lack of appropriate information, fear that the procedure will be painful, and healthcare staff attitudes perceived as being insensitive.[3, 26] The existence of such barriers can lead to a negative cancer screening experience, which might prevent disabled women from accessing preventive health services even if they are available.[4, 28]

One of the limitations of this study is that in the 2015 CASEN survey, disability was self-reported, which might have had an impact on validity and reliability. There is a substantial literature on the distinction between ‘subjective’, self-reported information, and information based on data, such as medical reports.[29] We found no information regarding response bias in the CASEN survey and how it was addressed.

The demographic changes in the Chilean society, with people living longer, mean that more people are likely to develop cancer; it is important that access to screening services is equitable so that some population groups are not disadvantaged.[7] An important step in this direction is moving beyond an exclusively single-disease approach to cancer management and develop approaches that take into account the complexity of the patient population and enable everybody to use screening services.[30]

## Conclusions

The study looked into the utilisation rates of the Papanicolaou test and the mammogram for disabled women in Chile. Despite recent health reforms aimed at increasing equity in access to healthcare, we found that disabled women were less likely to undergo these preventive tests than non-disabled women, and that a number of demographic, socioeconomic, and health-related factors affected utilisation. While in the last decade utilisation rates of preventive health services have increased for both disabled and non-disabled women, the gap in utilisation between the two groups has slightly increased. Taking into consideration demographic changes, it is imperative to address inequalities in access to preventive health services so as to improve health outcomes of disadvantaged groups, such as disabled women.
